# Seasonal Patterns of Enteric Pathogens in Colombian Indigenous People—A More Pronounced Effect on Bacteria Than on Parasites

**DOI:** 10.3390/pathogens11020214

**Published:** 2022-02-06

**Authors:** Simone Kann, Maria Hartmann, Juliane Alker, Jessica Hansen, Juan Carlos Dib, Andrés Aristizabal, Gustavo Concha, Ulrich Schotte, Lothar Kreienbrock, Hagen Frickmann

**Affiliations:** 1 Medical Mission Institute, 97074 Würzburg, Germany; simone.kann@medmissio.de; 2 Institute for Biometry, Epidemiology and Information Processing, Veterinary Medical University Hannover, 30559 Hannover, Germany; Maria.hartmann@tiho-hannover.de (M.H.); Lothar.kreienbrock@tiho-hannover.de (L.K.); 3 Department A-Veterinary Medicine, Central Institute of the Bundeswehr Medical Service Kiel, 24119 Kronshagen, Germany; juliane.alker@gmail.com (J.A.); U.Schotte@t-online.de (U.S.); 4 Department for Infectious Disease Diagnostics, Bernhard-Nocht Institute for Tropical Medicine, 20359 Hamburg, Germany; Jessica_hansen@gmx.de; 5 Tropical Health Foundation, Santa Marta 470003, Colombia; jdibdiaz@uninorte.edu.co (J.C.D.); ajaristizabal@fspt.co (A.A.); 6 Organization Wiwa Yugumaiun Bunkauanarrua Tayrona (OWYBT), Department Health Advocacy, Valledupar 200001, Colombia; gustavoconcha16@gmail.com; 7 Department of Microbiology and Hospital Hygiene, Bundeswehr Hospital Hamburg, 20359 Hamburg, Germany; 8 Institute for Medical Microbiology, Virology and Hygiene, University Medicine Rostock, 18057 Rostock, Germany

**Keywords:** seasonality, infectious gastroenteritis, tropics, Colombia, Indigenous, Wiwa, tropics, epidemiology

## Abstract

Enteric pathogens, which are frequently food- and waterborne transmitted, are highly abundant in Indigenous people living in remote rural areas of Colombia. As the frequency of gastroenteritis in the tropics shows seasonal differences, we analyzed variations of pathogen patterns in the stool samples of a Colombian Indigenous tribe called Wiwa during the dry (*n* = 105) and the rainy (*n* = 227) season, applying real-time PCR from stool samples and statistical analysis based on a multi-variable model. Focusing on bacterial pathogens, increased detection rates could be confirmed for enteropathogenic, enterotoxigenic and enteroaggregative *Escherichia coli* with a tendency for an increase in *Campylobacter jejuni* detections during the rainy season, while there was no seasonal effect on the carriage of *Tropheryma whipplei*. Salmonellae were recorded during the rainy season only. A differentiated pattern was seen for the assessed parasites. *Entamoeba histolytica*, *Necator americanus* and *Trichuris trichiura* were increasingly detected during the rainy season, but not *Ascaris lumbricoides*, *Giardia duodenalis, Hymenolepis nana*, *Strongyloides stercoralis*, and *Taenia solium*, respectively. Increased detection rates during the dry season were not recorded. Negative associations were found for *Campylobacter jejuni* and *Giardia duodenalis* with age and for *Tropheryma whipplei* with the body mass index, respectively. Positive associations of enteropathogenic *Escherichia coli* and *Taenia solium* detections were observed with age. In conclusion, facilitating effects of the tropical rainy season were more pronounced on bacterial enteric pathogens compared to enteropathogenic parasites.

## 1. Introduction

Seasonal effects on the epidemiology of infectious diseases in general and gastroenteric infections in particular are well-known phenomena. However, available information is still scarce and partly inhomogeneous. In an assessment with deployed European soldiers in the tropical South of Mali, particularly high gastroenteritis rates were observed in the moist, hot climate of the rainy season [1]. Decades before, U.S. American armed forces had a similar experience with seasonality of tropical gastroenteritis at a military base on the Philippines [2,3]. In the Colombian Indigenous Wiwa population, an increased rate of *Cyclospora cayetanensis* infections was observed in the rainy season as well [4], confirming an inconsistently reported seasonality of cyclosporiasis [5,6,7]. For other coccidian parasites like *Cryptosporidium* spp., similar associations with the rainy season have been reported from the tropics [8]. Increased rates of bacterial gastroenteritis due to pathogens like shigellae, enterotoxigenic *Escherichia coli* and *Vibrio cholerae* have been recorded in tropical Bangladesh in the hot and moist summer months [9]. In Sri Lanka, risk of *Campylobacter* spp.-associated diarrhea increased with increasing temperature and increasing humidity up to 80%, while further increases of humidity above 80% were associated with a reduction in this risk [10]. There are exemptions. *Clostridioides difficile* shows peaks of infection in spring in temperate climate zones [11], while the pathogen is comparably rarely detected in the tropics in human stool samples [12].

Interestingly, moisture-associated effects have also been described in the other direction. In tropical Northern Nigeria, Bangladesh and Costa Rica, increased rates of rotavirus-associated diarrhea have been predominantly recorded in the dry season [9,13,14,15], while the cool season in regions with temperate climate is predominantly associated with increased incidence rates irrespective of humidity or rainfall [14,16,17,18]. In subtropical Hong Kong, the colder winter season was also associated with increased rates of gastroenteritis due to human parechovirus [19], and in Taiwan, higher concentrations of adenovirus were recorded in river water in the winter months [20]. In addition, social reasons can contribute to seasonal disease peaks, as shown for the winter peak of *Campylobacter* spp.-associated diarrhea in Switzerland associated with meat fondues consumed during this festive season [21]. As confirmed by a study with diseased children in Singapore [22], the abovementioned observations suggest differentiated, species-dependent effects of climate conditions, resulting in no obvious effects of seasonality on all-cause gastroenteritis.

Next to infectious gastroenteritis, respiratory and febrile infections were also shown to be affected by seasonality in the tropics [23]. In children in tropical Singapore, both upper and lower respiratory tract infections were negatively associated with humidity [22]. Certain pathogens even obligatorily need special climate conditions like *Vibrio parahaemolyticus*, which requires warm, no-salinity water for transmission during the summer months [24], next to high inorganic phosphate concentration and copepod abundance [25].

In the Wiwa population in North-Eastern Colombia, high rates of infections or colonization with various gastrointestinal pathogens have been observed in the rainy season [26]. With a delay of about three years due to organizational and funding issues, the assessment was repeated in the dry season. In detail, the assessed population is at high risk for various gastrointestinal infections as their water sources are unprotected wells or cisterns as well as water directly taken from local rivers. In addition, there are no sanitary installations, and excrements are used as top dressing due to lack of awareness of the associated infection risks. The Wiwas live together with their livestock in limited space. Houses consist of clay and the roofs are made of palm tree leaves, which also contributes to many other health disorders such as Chagas disease, because the houses are optimal breeding places for various insects. Access to health care is sparse. Health points are rare and most of the time are not occupied, and if they are, then only with a community nurse. Infrastructure like roads is not in place; hospitals or better equipped health points are located on average 6 h walking distance away. Due to the Wiwas’ habitation of very remote areas, they are often not included in surveillance or health programs. According to weather information as provided by the company eglitis-media (Oldenburg, Germany), the averaged day temperature and night temperature are quite stable throughout the year with about 35 °C at daytime and about 25 °C at nighttime in the assessment region. In contrast, a doubling of the number of rainy days and an increase in the amount of rain by a factor of 3 to 4 can be expected when comparing the dry season assessment period January–April with the rainy season assessment period July–November. The aim of this study was the comparative assessment of pathogen patterns as observed in stool samples of the local population in tropical Colombia in the dry and rainy season in order to confirm hints for pathogen-specific seasonality.

## 2. Results

During the rainy season of 2014 (assessment period July–November 2014), 105 stool samples from the assessed Indigenous study population were included in the multi-variable model-based assessment, while 227 samples could be included from the dry season in 2018 (assessment period January–April 2018). A differentiated pattern with marked differences for bacteria and parasites could be shown.

Focusing on recorded detections of bacteria, all assessed diarrheagenic *Escherichia coli*, i.e., enteropathogenic, enterotoxigenic and enteroaggregative *E. coli*, were more frequently recorded in the rainy season than in the dry season. For *Campylobacter jejuni*, at least a non-significant tendency for more detections during the rainy season could be shown, while there was no effect on *Tropheryma whipplei*. *Salmonella* spp. (six records in the rainy season only), *Shigella* spp./enteroinvasive *E. coli* (two records in the rainy season, four records in the dry season), *Yersinia* spp. (no records at all) were too rarely detected to be included in the statistical assessment.

Focusing on the included parasites, increased frequency during the rainy season could be shown for the protozoon *Entamoeba histolytica* and the helminths *Necator americanus* and *Trichuris trichiura*. Thereby, the effect seen for *Entamoeba histolytica* was just based on very low numbers of seven detections in the rainy season and two detections in the dry season. In contrast, no seasonal effect could be shown for the protozoon *Giardia duodenalis* and the helminths *Ascaris lumbricoides*, *Hymenolepis nana*, *Strongyloides stercoralis*, and *Taenia solium*, respectively. The low detection rates for the protozoon *Cryptosporidium parvum* (one record in the rainy and dry season each) and the helminths *Ancylostoma* spp. (no records at all), *Enterobius vermicularis* (two records in the rainy season, four records in the dry season), *Schistosoma* spp. (one record in the dry season only), and *Taenia saginata* (no records at all), respectively, were insufficient for statistical assessments.

Details are provided in Table 1 and Table 2.

As indicated in the multi-variable model, a small number of recorded patient-specific features showed associations with pathogen detections. Thereby, the effects of additionally assessed parameters were strongly pathogen-depending. Of note, *Campylobacter jejuni* (Odds ratio (OR) 0.957, *p* = 0.0004) and *Giardia duodenalis* (OR 0.982, *p* = 0.0234) infections were negatively associated with patient age, enteropathogenic *Escherichia coli* (OR 1.031, *p* = 0.0005) and *Taenia solium* (OR 1.039, *p* = 0.0195) detections were positively associated with patient age, and body mass index (BMI) was negatively associated with *Tropheryma whipplei* (OR 0.795, *p* = 0.0091) detection, respectively. No other associations could be identified as statistically significant in the multi-variable model. In particular, there was no sex-specific association with pathogen detection and no association with gastrointestinal symptoms.

## 3. Discussion

The study aimed to address seasonality of different gastroenteric pathogens in the Indigenous tribe called Wiwa in North-Eastern Colombia. Seasonality of infectious gastroenteritis is not just an academic topic but an issue of practical relevance as demonstrated by considerable health care-associated costs in temperate climate zones, e.g., due to viral diarrhea outbreaks during the winter seasons [27]. In Japan, modellings of potential cost savings due to reduced numbers of inpatient gastroenteritis cases as likely beneficial consequences of global warming have been conducted [28], underlining the medical-economic relevance of the topic. In Indigenous Wiwa communities in North-Eastern Colombia, a differentiated pattern with a proportion of seasonality-associated effects deserving further discussion could be recorded in the present study, confirming previous findings with focus on *C. cayetanensis* [4].

Either more pathogen detections during the rainy season as compared to the dry season or no differences at all were recorded. A situation of higher proportions of gastrointestinal pathogen detections in the dry season was, in contrast, never observed. This is well in line with previous reports indicating increased disease activity of infectious gastroenteritis during the rainy season in tropical settings [1,2,3,4,5,6,7,8,9,10] and thus confirms those previous findings.

A highly pronounced seasonal effect on diarrheagenic coliform bacteria transmitted via the food- and water-borne route could be shown. In detail, this could be demonstrated for enteropathogenic, enterotoxigenic, and enteroaggregative *E. coli*, which are particularly associated with poor hygiene conditions, partly due to a lack of knowledge regarding appropriate hygiene procedures [29], partly due to lacking options of adequate hygiene procedures due to scarce resources. For *Salmonella* spp., low numbers of pathogen detections did not allow a sound statistical assessment. However, of note, Salmonella spp. detections were recorded in the rainy season only. For fecal-orally transmitted *Campylobacter jejuni* [30], only a tendency below the significance threshold could be recorded, but the trend was towards a higher infection rate in the rainy season in line with previous findings [10], potentially associated with higher humidity and reduced ultraviolet (UV) light exposure. For *Tropheryma whipplei*, no seasonal effect at all could be demonstrated. Direct nosocomial person-to-person-transmission, which is assumed to be a relevant mode of *T. whipplei*-transmission [31], shows little dependence on weather conditions, which is a likely explanation for the lacking seasonal effect.

A seasonal effect was much less pronounced and more species-dependent on enteric parasites compared to the observed effect on bacteria. So, the seasonal effect which was recently described for *C. cayetanensis* among the Wiwa population [4] could not be generalized. While no such effect could be demonstrated for the most assessed parasites, comprising locally frequently detected species like *Ascaris lumbricoides*, *Giardia duodenalis* and *Hymenolepis nana* [26], which are mainly transmitted by the consumption of contaminated food including raw meat in spite of hygiene training measures or close proximity to infected animals, there was a number of exemptions. So, increased frequency of detection during the rainy season could be shown for the soil-transmitted helminths *Necator americanus* and *Trichuris trichiura*, as well as for the water-borne protozoon *Entamoeba histolytica*. For the latter pathogen, however, the effect was calculated based on very few detections only, making an associated bias likely.

Apart from seasonal effects, a number of other associations with pathogen detections was shown to be statistically significant by the applied multi-variable model. Age-specific effects on *G. duodenalis* and *Campylobacter jejuni,* being more frequently observed in the stools of young children in the tropics with declining detection rates with increasing age, have previously been reported from tropical Africa as well [32,33]. Further, the pathogenic potential of enteropathogenic *E. coli* is believed to be restricted to young age [34], while the bacterium might become part of the gut flora with increasing age, potentially explaining the observed positive association with age.

Altogether, the calculations suggested just a minor influence of age. A stronger inverse association was recorded between the detection of *Tropheryma whipplei* and the calculated body mass index (BMI). This finding is interesting, because detections of *T. whipplei* in stool samples from the tropics have usually been considered as more or less harmless colonization due to inconsistent data on associations between *T. whipplei* in stool specimens and gastrointestinal symptoms in the tropics [35,36,37,38]. The here-observed association between low BMI and *T. whipplei* in the Wiwas’ stool samples, however, may speak in favor of potential health-related effects. Since the study was not designed to answer this question, future assessments should address the potential medical relevance of *T. whipplei* detections in the Wiwa population.

As recently indicated [4], the study has a number of limitations. First, the explorative design of the study in a population without certain information on prevalence rates and expected effect sizes did not allow a sample size calculation prior to the assessment based on reliable assumptions. Second, the time period of more than three years between the first and the second assessment, which was due to funding restrictions, makes influences other than seasonality possible. However, as the living conditions of the Wiwa population did not relevantly change in the meantime, only a minor influence of additional factors was assumed. Further, no specific events like protracted durations of the dry season or relevantly increased amounts of rain were reported for the years between the assessments. Third, the low and non-identical number of study participations makes the interpretation of the results challenging and any stratification for specific living conditions unfeasible. Fourth, the fact that no associations with reported gastrointestinal symptoms were found needs to be interpreted with care. As discussed before [4,26], complaints on gastroenteric disorders are socially discouraged in Wiwa populations, making an underreporting bias likely. Further, multiple pathogen detections in the same sample make conclusions on associations of symptoms with individual pathogens challenging.

## 4. Materials and Methods

### 4.1. Study Type

The study was conducted as a comparison of two cross sectional exploratory studies, of which the first was performed in the rainy season (July–November 2014) and the second in the dry season (January–April 2018) as an epidemiological follow-up assessment in Indigenous Wiwa populations living in a remote region of tropical Colombia, where high baseline colonization or infection rates with gastroenteric pathogens in the rainy season are known [26].

### 4.2. Study Population, Inclusion and Exclusion Criteria

As described before [4], the compositions of the two study populations were as shown in Table 2. Next to the laboratory assessments as listed below, recorded participant data comprised age, sex, height, weight, and self-reported gastroenteric symptoms (comprising abdominal pain or diarrhea) at the time of each sample collection. Sampling and pre-analytic conditions have been detailed elsewhere [4,26]. In short, diagnostic stool assessments with the option of subsequent therapy in case of pathogen detections were offered as a voluntary option for all Wiwas in the study area. Associated with the sample collection, the abovementioned participant data were recorded. Freshly collected stool samples were locally stored in cooling boxes and subsequently at −20 °C. After shipment to Germany, all samples were stored at −80 °C until further processing.

Data sets were included in the analysis if full information was available. Study participants without questionnaire-based information were excluded completely, whereas individually missing PCR results resulting from insufficient sample volumes were accepted, causing different sample sizes for different parameters. In compliance with these inclusion and exclusion criteria, the composition of the study populations was as shown in Table 3.

### 4.3. Diagnostic Procedures

To ensure comparability, the diagnostic assessments were identical for both study populations and performed as described recently [26,39,40,41,42,43,44]. In short, they comprised real-time PCR assessment of the stool samples targeting *Entamoeba histolytica, Giardia intestinalis, Cryptosporidium parvum, Necator americanus, Strongyloides stercoralis, Ascaris lumbricoides, Ancylostoma* spp., *Trichuris trichiura, Schistosoma* spp., *Enterobius vermicularis, Taenia saginata, Taenia solium, Hymenolepis nana*, *Campylobacter jejuni, Salmonella* spp., *Shigella* ssp./enteroinvasive *E. coli* (EIEC), *Yersinia* spp., enteropathogenic *E. coli* (EPEC), enterotoxin-producing *E. coli* (ETEC), enteroaggregative *E. coli* (EAEC), and *Tropheryma whipplei*. Nucleic acid extraction was performed with the QiaAMP DNA Stool Mini Kit (Qiagen, Hilden, Germany) according to the manufacturer’s protocol, eluates were stored at −80 °C prior to the PCR assessments. All real-time PCR protocols, including positive, negative and extraction controls, were run exactly as published [39,40,41,42,43,44] and diagnostically provided by our laboratory applying the HotStar master mix (Qiagen, Hilden, Germany) as reaction chemistry on RotorGene Q cyclers (Qiagen, Hilden, Germany). The detailed PCR protocols have been described elsewhere [40,41,42,44], a summary of the oligonucleotide sequences and target genes is provided as Table A1.

### 4.4. Statistics

All data were analysed using the software SAS, version 9.4 TS Level 1M5. For data description and unifactorial comparison purposes, univariable logistic regression including Wald’s test was used for both categorical and continuous data to compare negative with positive test-outcomes. To test for homogeneity between the studies in 2014 and 2018, Wald’s test for categories and non-adjusted Wilcoxon-testing only for the category “PCR-positive” were applied. To ensure a multi-factorial perspective on the data and to control for potential confounding effects, a multivariable logistic regression was utilized for those factors which appeared statistically significant on the univariable level. Statistical significance was defined as *p* < 0.05.

### 4.5. Ethics

Ethical clearance for the assessment of the population in 2014 was provided by the Ethics Committee of Valledupar, Cesar, Colombia (Acta no 0022013, February 2013), for the assessment of the population in 2018 by the Ethics Committee for Research in Santa Marta (Acta no 102016, October 2016). From each participant or from the parent or legal guardian of children, written informed consent was obtained prior to study participation. Agreement with the principles of the Declaration of Helsinki was assured for the study.

## 5. Conclusions

In spite of the above-mentioned limitations, the study indicated that the tropical rainy season is associated with increasing detection rates of enteric pathogens in the Wiwa population. Increased moisture associated with temperatures of about 35 °C provides optimized growth conditions for microorganisms and livestock lives even more closely associated with the Wiwas in the rainy season. Higher detection rates in the dry season were not observed. Increased detection rates were more pronounced for water-borne bacterial pathogens with particular emphasis on diarrheagenic *Escherichia coli*. For enteric parasites, in contrast, a differentiated pattern of increased and unchanged detection rates was observed.

## Figures and Tables

**Table 1:** Seasonal distribution of pathogens as included in the assessment ordered by group and quantitative relevance.

	Screening in the Rainy Season * of 2014 (*n* = 105)		Screening in the Dry Season * of 2018 (*n* = 227)	
Assessed Microorganism	Positive, *n* (%)	Negative, *n* (%)	Positive, *n* (%)	Negative, *n* (%)
Bacteria (in the order of total numbers of detections within the study)				
Enteropathogenic *Escherichia coli* (EPEC)	45 (42.9%)	60 (57.1%)	65 (28.6%)	162 (71.4%)
Enteroaggregative *Escherichia coli* (EAEC)	69 (65.7%)	36 (34.3%)	40 (17.6%)	187 (32.4%)
*Campylobacter jejuni*	31 (29.5%)	74 (70.5%)	48 (22.2%)	168 (77.8%)
Enterotoxigenic *Escherichia coli* (ETEC)	44 (41.9%)	61 (58.1%)	25 (11.0%)	202 (89.0%)
*Tropheryma whipplei*	7 (6.7%)	98 (93.3%)	12 (5.3%)	215 (94.7%)
*Salmonella* spp.	0 (0.0%)	105 (100.0%)	6 (2.6%)	221 (97.4%)
*Shigella* spp./enteroinvasive *Escherichia coli* (EIEC)	2 (1.9%)	103 (98.1%)	4 (1.8%)	225 (98.2%)
*Yersinia* spp.	0 (0.0%)	105 (100.0%)	0 (0.0%)	227 (100.0%)
Protozoa (in the order of total numbers of detections within in the study)				
*Giardia intestinalis*	50 (57.6%)	55 (52.4%)	117 (54.2%)	99 (45.8%)
*Entamoeba histolytica*	7 (6.7%)	98 (93.3%)	2 (0.9%)	214 (99.1%)
*Cryptosporidium parvum*	1 (0.9%)	104 (99.1%)	1 (0.4%)	226 (99.6%)
Helminths (in the order of total numbers of detections within in the study)				
*Hymenolepis nana*	21 (20.0%)	84 (80.0%)	43 (18.9%)	184 (81.1%)
*Necator americanus*	20 (19.0%)	85 (81.0%)	22 (10.2%)	194 (89.8%)
*Trichuris trichiura*	23 (21.9%)	82 (78.1%)	12 (5.3%)	215 (94.7%)
*Ascaris lumbricoides*	11 (10.5%)	94 (89.5%)	21 (9.7%)	195 (90.3%)
*Taenia solium*	6 (5.7%)	99 (94.3%)	4 (1.8%)	223 (98.2%)
*Strongyloides stercoralis*	3 (2.9%)	102 (97.1%)	6 (2.8%)	210 (97.2%)
*Enterobius vermicularis*	2 (1.9%)	103 (98.1%)	4 (1.8%)	225 (98.2%)
*Schistosoma* spp.	0 (0.0%)	105 (100.0%)	1 (0.4%)	226 (99.6%)
*Ancylostoma* spp.	0 (0.0%)	105 (100.0%)	0 (0.0%)	227 (100.0%)
*Taenia saginata*	0 (0.0%)	105 (100.0%)	0 (0.0%)	227 (100.0%)

* Rainy season: assessment period July–November 2014. Dry season: assessment period January–April 2018.

**Table 2:** Odds ratios and significance levels for the different pathogen detections. Only pathogen detections were included for which sufficient numbers for a sound calculation were recorded in both seasons.

Pathogens	More Detections in the Rainy Season Than in the Dry Season		Male Versus Female (Reference)		Gastrointestinal Symptoms		Age Per 1 Year		Body Mass Index per 1 kg/m^2^	
Ordered by Bacteria, Protozoa and Helminths	Odds Ratio in the Multimodal Model	Significance (Threshold 0.05)	Odds Ratio in the Multimodal Model	Significance (Threshold 0.05)	Odds Ratio in the Multimodal Model	Significance (Threshold 0.05)	Odds Ratio in the Multimodal Model	Significance (Threshold 0.05)	Odds Ratio in the Multimodal Model	Significance (Threshold 0.05)
Enteropathogenic *Escherichia coli* (EPEC)	1.943	0.0104	0.855	0.5271	1.481	0.2433	1.031	0.0005	0.935	0.0582
Enteroaggregative *Escherichia coli* (EAEC)	9.513	<0.0001	0.648	0.1182	1.233	0.5862	1.009	0.3379	1.009	0.7910
*Campylobacter jejuni*	1.735	0.0601	1.181	0.5459	0.700	0.4336	0.957	0.0004	1.017	0.6635
Enterotoxigenic *Escherichia coli* (ETEC)	6.130	<0.0001	0.928	0.8005	0.947	0.8989	0.998	0.8485	0.990	0.7890
*Tropheryma whipplei*	1.975	0.2136	2.588	0.0795	0.918	0.9029	1.007	0.6574	0.795	0.0091
*Giardia intestinalis*	0.810	0.3918	1.274	0.2968	0.949	0.8789	0.982	0.0234	0.989	0.7109
*Entamoeba histolytica*	10.862	0.0057	0.247	0.0952	0.836	0.8828	0.996	0.8909	0.867	0.2418
*Hymenolepis nana*	1.063	0.8426	1.255	0.4263	0.849	0.7002	0.988	0.2471	1.012	0.7513
*Necator americanus*	2.118	0.0279	0.624	0.1759	0.786	0.6511	1.009	0.3863	0.986	0.7592
*Trichuris trichiura*	5.411	<0.0001	1.259	0.5521	0.763	0.6500	0.992	0.5409	0.948	0.3428
*Ascaris lumbricoides*	1.177	0.6855	0.593	0.1795	0.911	0.8721	1.004	0.7310	0.950	0.3514
*Taenia solium*	2.889	0.1169	0.838	0.7950	1.502	0.6115	1.039	0.0195	0.949	0.5449
*Strongyloides stercoralis*	0.941	0.9334	1.372	0.6475	1.604	0.5792	1.012	0.5283	1.028	0.7175

**Table 3:** Composition of both study populations.

	Study Population 1 (Rainy Season, July–November 2014)				Study Population 2 (Dry Season, January–April 2018)			
Village (Number (*n*) of individuals assessed with coverage in %)	*n*		coverage of the entire population in %		*n*		coverage of the entire population in %	
Tezhumake, Department Cesar	6		27.6		84		33.6	
Siminke, Department La Guajira	3		20.0		25		16.1	
Valledapur, Department Cesar	5		0.01		76		63.3	
Ashintukwa, Department La Guajira	n.a.		n.a.		42		16.8	
All	105				227			
Sex (*n*, %)	*n*		%		*n*		%	
Female	54		51.4		114		50.2	
Male	51		48.6		113		49.8	
Gastrointestinal symptoms (*n*, %)	*n*		%		*n*		%	
No	89		84.8		194		85.5	
Yes	16		15.2		33		14.5	
Age in years	*n*	mean	median	STD	*n*	mean	median	STD
	105	24.5	20.0	18.0	227	19.6	10.6	18.0
Body Size in cm	*n*	mean	median	STD	*n*	mean	median	STD
	104	136.0	144.0	24.4	225	124.8	125.0	25.9
Weight in kg	*n*	mean	median	STD	*n*	mean	median	STD
	103	41.7	45.0	16.2	226	33.9	26.7	19.0
Body mass index (BMI) in kg/m^2^	*n*	mean	median	STD	*n*	mean	median	STD
	103	21.5	20.8	3.9	225	19.9	18.2	5.0

STD = standard deviation. n.a. = not applicable.

## Data Availability

All relevant data are provided in the manuscript or the Appendix tables. Raw data can be made available on reasonable request.

## Appendix A

**Table A1:** Target genes as well as sequences of the oligonucleotides used for the real-time PCR assessments (in alphabetic order) and references including the detailed protocols.

Target Pathogen	Target Gene	Forward Primer Sequence	Reverse Primer Sequence	Probe Sequence	Reference, where the Detailed Protocol Can Be Found
*Ancylostoma* spp.	ITS2	GAATGACAGCAAACTCGTTGTTG	ATACTAGCCACTGCCGAAACGT	ATCGTTTACCGACTTTAG	[40]
*Ascaris lumbricoides*	ITS1	GTAATAGCAGTCGGCGGTTTCTT	GCCCAACATGCCACCTATTC	TTGGCGGACAATTGCATGCGAT	[40]
*Campylobacter jejuni*	*gyrA*	CTATAACAACTGCACCTACTAAT	AAGTGTAAGCACACAAGGTA	CTTAATAGCCGTCACCCCAC	[44]
*Cryptosporidium parvum*	138-bp fragment inside the *C. parvum*-specific 452-bp fragment	CGCTTCTCTAGCCTTTCATGA	CTTCACGTGTGTTTGCCAAT	CCAATCACAGAATCATCAGAATCGACTGGTATC	[40]
*Entamoeba histolytica*	SSU rRNA gene	ATTGTCGTGGCATCCTAACTCA	GCGGACGGCTCATTATAACA	TCATTGAATGAATTGGCCATTT	[40]
*Enterobius vermicularis*	ITS1	CGGTGTAATTTTGTTGGTGTCTATG	TGGCAGCATTGCAAACTAATG	TGTGCCAGTCAACGCCTAAACCGTC	[40]
*Escherichia coli*, enteroaggregative (EAEC)	*aatA*	CAATGTATAGAAATCCGCTGTT	CTGTCAGATAAAATCTCGAGAGAA	CATGTTCCTGAGAGTGCAATCCCAG	[42]
*Escherichia coli*, enteropathogenic (EPEC)	*eae*	CATTGATCAGGATTTTTCTGGTGATA	CTCATGCGGAAATAGCCGTTA	ATAGTCTCGCCAGTATTCGCCACCAATACC	[42]
*Escherichia coli*, enterotoxigenic (ETEC)	*eltB*	GCGTTACTATCCTCTCTATG	TGATATTCCGAACATAGTTCTGTA	TAGACTGGGGAGCTCCGTGTGC	[42]
	*estB*	TCCCTCAGGATGCTAAAC	CAACAAAGCAACAGGTACATACGT	ATAGCACCCGGTACAAGCAGG	[42]
*Giardia intestinalis*	SSU rRNA gene	GACGGCTCAGGACAACGGTT	TTGCCAGCGGTGTCCG	CCCGCGGCGGTCCCTGCTAG	[40]
*Hymenolepis nana*	ITS1	CATTGTGTACCAAATTGATGATGAGTA	CAACTGACAGCATGTTTCGATATG	CGTGTGCGCCTCTGGCTTACCG	[40]
*Necator americanus*	ITS2	CTGTTTGTCGAACGGTACTTGC	ATAACAGCGTGCACATGTTGC	CTGTACTACGCATTGTATAC	[40]
*Salmonella* spp.	*ttrC*	ATTGTTGATTCAGGTACAAAC	AATTAGCCATGTTGTAATCTC	CAAGTTCAACGCGCAATTTA	[44]
*Schistosoma* spp.	ITS2	GGTCTAGATGACTTGATYGAGATGCT	TCCCGAGCGYGTATAATGTCATTA	TGGGTTGTGCTCGAGTCGTGGC	[40]
*Shigella* spp./*Escherichia coli*, enteroinvasive (EIEC)	*ipaH*	CAGAAGAGCAGAAGTATGAG	CAGTACCTCGTCAGTCAG	ACAGGTGATGCGTGAGACTG	[44]
*Strongyloides stercoralis*	18S rRNA gene	GAATTCCAAGTAAACGTAAGTCATTAGC	TGCCTCTGGATATTGCTCAGTTC	ACACACCGGCCGTCGCTGC	[40]
*Taenia saginata*	ITS1	GCGTCGTCTTTGCGTTACAC	TGACACAACCGCGCTCTG	CCACAGCACCAGCGACAGCAGCAA	[40]
*Taenia solium*	ITS1	ATGGATCAATCTGGGTGGAGTT	ATCGCAGGGTAAGAAAAGAAGGT	TGGTACTGCTGTGGCGGCGG	[40]
*Trichuris trichiura*	18S rRNA gene	TTGAAACGACTTGCTCATCAACTT	CTGATTCTCCGTTAACCGTTGTC	CGATGGTACGCTACGTGCTTACCATGG	[40]
*Tropheryma whipplei*	*Dig 15*	TGTTTTGTACTGCTTGTAACAGGATCT	GATGATAGGAGGGATAGA-GCAGGA	AGAGATACATTTGTGTTAGTTGTTACA	[41]
	*Dig 15*	TGAGTGATGGTAGTCTGAGAGATATGT	TCCATAACAAAGACAACAACCAATC	AGAAGAAGATGTTACGGGTTG	[41]
*Yersinia* spp.	*ail*	GCATTAACGAATATGTTAGC	ATCGAGTTTGGAGTATTCAT	CCGCTTCCAAATTTTGTCAT	[44]

bp = base pairs, ITS = internal transcribed spacer, rRNA = ribosomal ribonucleic acid, SSU = small subunit.
